# Dietary Phytogenic Combination with Hops and a Mixture of a Free Butyrate Acidifier and Gluconic Acid Maintaining the Health Status of the Gut and Performance in Chickens

**DOI:** 10.3390/ani10081335

**Published:** 2020-08-02

**Authors:** Katarzyna Ząbek, Dominika Szkopek, Monika Michalczuk, Paweł Konieczka

**Affiliations:** 1 Department of Animal Nutrition, Kielanowski Institute of Animal Physiology and Nutrition, Polish Academy of Sciences, Instytucka 3, 05-110 Jabłonna, Poland; k.zabek@ifzz.pl (K.Z.); d.szkopek@ifzz.pl (D.S.); 2 Department of Animal Breeding, Institute of Animal Sciences, Warsaw University of Life Sciences, Ciszewskiego 8, 02-786 Warsaw, Poland; monika_michalczuk@sggw.edu.pl; 3 Department of Poultry Science, University of Warmia and Mazury in Olsztyn, 10-719 Olsztyn, Poland

**Keywords:** broiler chicken, prebiotic, phytogenic ingredients, acidifier, microbiota activity, gut health

## Abstract

**Simple Summary:**

Throughout the rearing period of broiler chickens, they are exposed to a number of stress factors that may compromise their health status. It is particularly important that the development of the gut with a balanced microbiome is not disturbed, which determines the overall health of the birds to a large extent. A number lines of evidence have indicated that substances with bioactive properties, including prebiotics, organic acids, and/or phytogenic ingredients of plants, may have beneficial actions in maintaining the good health status of the bird gut without compromising performance. In the present study, we investigated the dietary application of phytogenic ingredients from hops, licorice and arabic gum as well as a free butyrate acidifier together with a prebiotic complex in relation to the gut functional status response of the host. The present report confirmed that feeding the birds with the dietary treatments maintained bird growth and normal healthy status of the gut and improved gut morphostructure indices. Our findings indicate the potential of the investigated additives for modulating gut functional status in birds.

**Abstract:**

Additives with bioactive properties can improve chickens’ gut health. This study investigated the physiological status of the gut, including its morphological structure and microbiome activities in chickens fed diets supplemented with phytogenic ingredients with hops (Anta^®^Phyt) or a mixture of a free butyrate acidifier and gluconic acid (PreAcid). In this study, 1155 broilers were distributed to three dietary treatments with 5 replicate pens per treatment, 77 birds each. Anta^®^Phyt was added at 400/300/200/200 mg/kg diet whereas PreAcid was added at 3/2/1/1 g/kg starter/grower I/grower II/finisher diet respectively. Dietary treatments did not compromise body weight in different growth periods. In the birds fed PreAcid-supplemented diet, higher gut concentration of butyric acid was observed, particularly in the early stage of growth, while the profile of the short-chain fatty acids was maintained among the treatments. Neither additive significantly affected cecal bacterial enzyme activities. Feeding the birds with Anta^®^Phyt and PreAcid had beneficial effects on gut morphostructure indices, including intestinal wall thickness, crypt depth and the villus height to crypt depth ratio, in 35- and 42-day old birds. In conclusion, the feeding Anta^®^Phyt- or PreAcid-supplemented diet exerted beneficial effects on the indices determining the physiological status of the gut and maintained good performance of birds of different ages.

## 1. Introduction

The bird digestive tract is a large reservoir of bacterial communities that colonize the intestinal mucosa. The presence of these organisms is important for the proper functioning of the whole biological system. The composition of the microflora in the digestive tract is constantly changing, but the balance between microorganisms that are beneficial to the host and pathogens is important because it determines the health functional status of the gut. There are a number of stress factors during the rearing period that can cause an imbalance in the gut microflora, leading to health abnormalities.

A healthy digestive tract results in better digestion, lower nutrient losses, better growth, health, and pathogen resistance and lower mortality. Feed additives such as phytogenics, acidifiers, or prebiotics have a positive effect on intestinal function [1,2,3,4,5,6,7]. Prebiotics modulate the composition and growth of beneficial microflora in the gastrointestinal tract, thus preventing the development of pathogenic organisms. There is a great deal of evidence that prebiotics can affect the gut microbiome of birds but, still the mechanisms of their action warrant deeper investigation [1]. One of the many substances used as a prebiotic is gluconic acid. This organic acid, produced through the fermentation of starch and can be added to the feed, can be converted to butyrate, which can in turn be used as an energy source for colonocyte and bacterial proliferation in the host gut [2]. Similarly, butyric acid is a source of energy for intestinal epithelial cells and improves their growth, regulates the intestinal blood supply and mucus production and stimulates local immunity [3,4]. These properties make butyric and gluconic acid to feed additives (prebiotics and acidifiers) with supportive effects on gut function.

It has been reported that phytogenics show also the potential to beneficially influence on the chicken’s gastrointestinal tract [5,6,7]. Phytogenics are preparations containing parts and extracts of various plants with known beneficial effects on organisms. Licorice or hops are additives of potential interest that are reported to have properties that contribute to the development of a favorable environment for beneficial bacterial proliferation in the broiler gut [8,9,10]. Therefore, additives that can contribute to good intestinal health status while maintaining bird performance indices are currently of high interest for the poultry sector.

The effect of various phytogenic compounds and organic acid additives have been investigated toward performance improvement of broilers [11]. However, data regarding the gut response in chickens are still scarce due to a complex host gut and nutrients of bioactive properties interactions. The current study investigated the physiological status of the gut, including morphological structure, absorptive potential of villi and gut microbial activity in broiler chickens of different ages fed diets supplemented with phytogenic ingredients with hops or a mixture of a free butyrate acidifier and gluconic acid.

## 2. Materials and Methods

### 2.1. Birds, Diets, and Experimental Design

A total of 1155 day-old male broilers of the Ross 308 strain purchased from a local commercial hatchery were randomly distributed to three dietary treatments with 5 replicate pens per treatment, each consisting of 77 birds. Chickens were initially vaccinated against the following diseases: Gumboro, Marek’s, Newcastle, and infectious bronchitis, using a commercial vaccination program. Three soybean-meal-, wheat-, and maize-based diets were prepared and were fed to the chickens ad libitum according to the following feeding program: Starter—days 1–10; grower I—days 11–24; grower II—days 25–35; and finisher—days 36–42. The diets were isoproteic and isocaloric, and each feeding phase was formulated to meet or exceed the requirements for Ross 308 broiler chickens [12]. The chickens were fed a control diet (CON); the control diet supplemented (on top) with phytogenic ingredients from hops, licorice and arabic gum (Anta^®^Phyt) at the following doses: 400 mg/kg diet in the starter phase, 300 mg/kg diet in the grower I phase, 200 mg/kg diet in the grower II phase and 200 mg/kg diet in the finisher phase; or a diet supplemented with a mixture of a free butyrate acidifier and gluconic acid (PreAcid) at the following doses: 3 g/kg diet in the starter phase, 2 g/kg diet in the grower I phase and 1 g/kg diet in grower II phase and finisher phase. Anta^®^Phyt contained more than 50% hop ingredients in combination with a premix of licorice and arabic gum, whereas PreAcid contained 65% organic acids (formic, lactic) and their salts and 30% prebiotic complex, according to the manufacturer’s declaration. The starter diets were fed to the birds as crumbles, whereas the grower- and finisher-type diets were cold pelleted in our laboratory in a CL-2 CPM (California Pellet Mill Equipment, Crawfordsville, IN, USA) laboratory pellet mill. The conditions of the room in which the chickens were housed were maintained according to standard management practices for commercial chicken houses.

The experiment was conducted under a randomized block design, with each group of birds assigned to one of 3 treatments.

All procedures in the present study were performed in accordance with the principles of the European Union and Polish Law on Animal Protection.

### 2.2. Sampling Procedures

On days 28, 35, and 42 of age, chickens were weighed, and a total of 12 chickens from each dietary treatment that were representative of the mean group BW were selected, electrically stunned and sacrificed by decapitation. The luminal contents of the cecum of each bird were gently squeezed into sterile tubes and pooled to provide sufficient material for analyses. Subsequently, approximately 5 g of digesta was portioned into sterile test tubes (in three replicates) and stored at −32 °C until analysis. The respective cecal samples were subjected to the determination of the following indices (*n* = 12 for each determination): Bacterial enzyme activity and concentration of short-chain fatty acids (SCFAs). From the same birds, samples of the duodenum, middle jejunum, and middle ileum were collected, cleaned of digesta by flushing with 0.9% saline, and fixed in a 4% formaldehyde solution for the evaluation of intestinal morphostructures.

### 2.3. Analyses of Caecal Microbiota Activity

The activity of five enzymes in the cecal digesta, including α- and β-glucosidase, α- and β-galactosidase, and β-glucuronidase, was measured based on the amounts of *p*-nitrophenol (PNP) or *o*-nitrophenol (ONP) released from the corresponding nitrophenylglucoside substrates as previously described [13]. Enzyme activity was measured spectrophotometrically (Multiskan Sky Microplate Spectrophotometer, Thermo Fisher Scientific, Waltham, MA, USA) and was expressed as the micromoles of product formed per minute (U) per gram of sample. The p-nitrophenol concentration was measured according to its optical absorbance at 400 nm, while o-nitrophenol was quantified at 420 nm. Each sample of digesta was analyzed in triplicate in separate runs.

In addition to enzyme activity analysis, cecal fermentation processes were also analyzed based on the concentration of SCFAs. The SCFA concentration in the cecal digesta was determined by gas chromatography (GC) on a GC instrument (Hewlett Packard, Waldbronn, Germany) equipped with a Supelco Nukol fused silica capillary column (30 m × 0.25 mm internal diameter, 0.25 mm film), using isocaproic acid as an internal standard according to a modified protocol from Konieczka et al. [14]. Briefly, after collection, the digesta samples were converted into their respective sodium salts by adjusting the pH to 8.2 with a 1 M NaOH solution. Twelve hours before the analysis, the samples were thawed at 4 °C and then centrifuged at 10,000× *g* for 10 min. The following GC conditions were applied: The initial temperature of the oven was kept at 100 °C for 2 min, followed by heating at 10 °C per 10 min to 140 °C and holding for 20 min. The injector temperature was maintained at 220 °C, while the detector was maintained at 250 °C. All analyses were performed in duplicate.

### 2.4. Analysis of Gut Morphostructure Indices

Samples of the duodenum, jejunum and ileum collected for morphometry evaluation were processed according to a previously described protocol [15]. After dehydration, the samples were embedded in paraffin wax and were then cut into transverse sections (4.5 µm) on a liquid microtome. Sections mounted on silanized slides were stained with hematoxylin and eosin for microscopy evaluation. Gut morphostructure parameters including villus height (VH) (measured from the tip of the villus to the villus-crypt junction), crypt depth (CD) (measured from the crypt mouth to the base), villus width (VW) (measured at the midline of the villus), the thickness of the intestinal wall (WT), the villus perimeter (calculated as (2π × (average villus width/2) × VH)) and the villus surface area (VA) (calculated as villus perimeter × VH) were measured. Measurements were taken only from sections where the section plane showed a vertical orientation (averages represent at least two slides with a minimum of 20 well-oriented indices).

### 2.5. Statistical Analysis

The experiment was performed under a completely randomized design. Data variability was expressed as the pooled standard error of the mean (SEM), and *p <* 0.05 was considered statistically significant. Differences between groups were determined using one-way ANOVA with the least significant difference (LSD) test. Statistical calculations were performed using STATGRAPHICS Centurion XVI ver. 16.1.03 software.

### 2.6. Ethical Statement

Qualified veterinarians performed all procedures involved handling the birds. No action involving pain or suffering was practiced, and all of the analysis were performed on samples collected post-mortem. The protocol for this study and the number of chickens used in this study were consistent with the regulations of the Local Committee for Experimentation on Animals (Warsaw, Poland, resolution no. WAW2/174/2019) and were performed in accordance with the principles of the European Union Directive 2010/63/EU for animal experiments and Polish Law on Animal Protection.

## 3. Results and Discussion

### 3.1. Bird Performance

**Figure 1:**
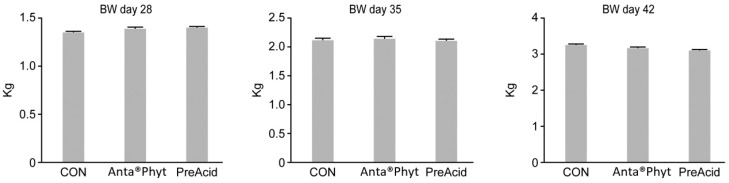
The effect of the dietary treatments on the body weight (BW) of chickens at days 28, 35 and 42 of age. Birds were fed a control diet (CON); the control diet supplemented (on top) with phytogenic ingredients from hops, licorice and arabic gum (Anta^®^Phyt); or a diet supplemented with a mixture of a free butyrate acidifier and prebiotic gluconic acid (PreAcid). Data are the means ± SEMs of 12 replicates per treatment. Differences between groups were considered significant when *p* < 0.05.

The mean final BWs of the birds at days 28, 35, and 42 averaged 1.38 ± 0.08, 2.13 ± 0.06, and 3.18 ± 0.07 kg respectively (Figure 1). Before sampling, birds were weighted at days 1, 10 and 24. The BWs at the respective periods averaged 43.01 ± 0.112, 305.0 ± 18.23, and 1249.1 ± 71.0 g (data not shown). In each investigated period, BW did not differ significantly between the dietary treatments (*p* > 0.05). The obtained data demonstrate that the diets containing either Anta^®^Phyt or PreAcid were equally well utilized by the birds, and BW was within the expected range for Ross 308 male broilers of a similar age [12]. In contrast, Hu and Guo [16] showed that dietary sodium butyrate supplementation increased BW gain in the period from 1 to 21 days in broilers. Jacobs and Parsons [17] observed that feeding chickens a 30 g/kg diet of gluconic acid compromised BW gain. The authors speculated that this could have been due to an increase in passage through the gastrointestinal tract, which might contribute to lower nutrient utilization. Our findings are in accordance with those presented by Midilli et al. [18], who reported that prebiotic supplementation did not significantly affect chicken BW, whereas other reports have demonstrated that different prebiotics may improve growth performance [19,20]. The possible disparities among the reports of bird responses could be due to differences in the bird strain, age, or sex, the nutrient composition of the diet, the gut microbiome activity response, the level of additive inclusion, the duration of supplementation and/or other environmental and management conditions between the studies. Grashorn [21] analyzed data on the use of phytogenic products in broiler nutrition from the literature. The plants included in phytogenic products vary widely in the composition, concentration and/or bioavailability to the host of their active ingredients. This indicates that the specific actions of different bioactive compounds in herbal additives cannot be easily distinguished from each another and that the study of their potential activity using a broiler model is warranted.

### 3.2. SCFA Concentrations and Profile

The results regarding the SCFA concentrations in the cecal digesta are shown in Table 1. The concentration of acetic acid in the birds sampled at day 28 was similar under the CON and Anta^®^Phyt treatments but was significantly higher under the PreAcid treatment than the Anta^®^Phyt treatment (*p =* 0.042). The same was true regarding butyric acid; feeding the birds the PreAcid-treated diet resulted in a significantly higher concentration of butyric acid in the cecal digesta (*p =* 0.04). The concentration of valeric acid in the cecal digesta was highest in the birds fed the PreAcid-treated diet (*p =* 0.049). These differences contributed to the total SCFA concentration, which was significantly higher under this treatment and did not differ between CON and Anta^®^Phyt (*p =* 0.022). In the birds sampled at day 35 of age, the dietary treatments did not significantly affect either the concentrations of each single SCFA or the total SCFAs (*p >* 0.05). However, the dietary treatments affected the profile of the most abundant SCFAs in the cecal digesta. The acetic acid concentration was highest in the birds fed CON, followed by those fed the PreAcid diet, and was lowest in those fed Anta^®^Phyt (*p <* 0.001). The butyric acid share in the SCFA profile was significantly higher in the Anta^®^Phyt treatment than in the other groups *(p =* 0.006). In birds sampled at 42 days of age, there were no significant differences regarding either concentrations of particular SCFAs or in their profile (*p >* 0.05).

SCFAs are the major end products of bacterial metabolism in broilers. Acetic, propionic, and butyric acids are mainly formed through the catabolism of carbohydrates and amino acids, whereas valeric, iso-butyric, and iso-valeric acids are formed from branched-chain amino acids. Therefore, a particularly important finding of the present study is that the dietary treatments maintained or promoted the production of butyric acid in the gut, which was mostly evident in the birds fed the PreAcid-supplemented diet in the early stage of their growth. This could be a consequence a prebiotic-action of gluconate in PreAcid additive being manifested in endogenous formation of butyric acid in the gut. Butyric acid is absorbed by the intestinal epithelium and stimulates its growth and regulates mucin production and intestinal immune functions [22]. It has also been reported that acetic acid, which is the most abundant SCFA in the intestinal digesta, can be used as a precursor for the synthesis and accumulation of butyric acid [23]. One of the possible mechanisms whereby the dietary treatments affected SCFAs in cecal digesta might have been the alteration of the gut pH. According to other reports, the addition of organic acids, including butyric, formic, and propionic acids, to either water [24] or feed [25] lowers not only the pH of the digesta but also its viscosity and moisture and affects nutrient absorption. Nevertheless, based on our previous studies [26,27], it can be concluded that the applied treatments were unlikely to be associated with disturbances in the fermentation process and even slightly improved it, which was manifested in the increase in the butyric acid concentration in the cecum digesta. In fact, acetic, propionic and butyric acids, which showed the highest abundance in the broiler cecum SCFA profile, were also present at the highest abundance in the birds subjected to different dietary treatments, which indicate a health-promoting effect on the broiler gut physiological status.

### 3.3. Bacterial Enzyme Activity

**Figure 2:**
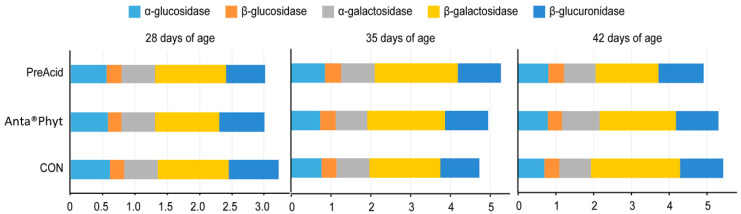
The effect of dietary treatments on the bacterial enzyme activities (μmol/h/g) in the cecal digesta of chickens at days 28, 35 and 42 of age. Birds were fed a control diet (CON); the control diet supplemented (on top) with phytogenic ingredients from hops, licorice and arabic gum (Anta^®^Phyt); or a diet supplemented with a mixture of a free butyrate acidifier and prebiotic gluconic acid (PreAcid). Data are the means of 12 replicates per treatment. Differences between groups were considered significant when *p <* 0.05.

The activities of bacterial enzymes in the broiler cecum are considered to be biomarkers of dietary ingredient–host interactions. In the present study, analysis of bacterial enzyme activities in the cecal digesta (Figure 2) revealed that α- and *β*-glucosidase, α- and *β*-galactosidase, and *β*-glucuronidase activities were not significantly affected by either the dietary treatments or the respective ages of the birds (*p >* 0.05). The negligible response of bacterial enzyme activities reported in the present study indicates that the two additives likely did not affect the adaptation of the gut microbiome to higher energy uptake, which could have resulted in a cost to the host and would be manifested in BW depression. According to a review by Zduńczyk et al. [28], feeding chicken diets with different prebiotics and/or butyric acid additives is usually associated with increased bacterial activities (higher activities of cecal enzymes and increased production of SCFAs) in the lower parts of the gut, which in turn is manifested in compromised performance of birds.

### 3.4. Gut Morphostructure Indices

The changes in gut mucosa morphostructure resulting from the dietary treatments are presented in Table 2, Table 3 and Table 4. The dietary treatments slightly affected the morphometry indices of different segments of the broiler gut. In the birds at 28 days of age, the dietary treatments did not affect gut morphostructures (*p >* 0.05). In older birds, the crypt depth in the duodenum was greater in the group subjected to the PreAcid treatment (*p =* 0.005), while villus width was greater in the control group (*p <* 0.001), which resulted in the greatest villus area in this group (*p =* 0.011). In the jejunum of the 35-d-old birds, crypt depth was greatest in the birds fed the PreAcid diet (*p <* 0.001), while wall thickness was significantly greater under the Anta^®^Phyt and PreAcid treatments than in the control (*p =* 0.004). In the ileum, only villus width was affected by the dietary treatments, which was significantly narrower under the PreAcid treatment than in the control (*p =* 0.048). However, this difference did not result in significant differences in the surface area of the villi (*p >* 0.05). In the birds at 42 days of age, the dietary treatments affected villus width, which was significantly greater in the duodenum in the control than under the Anta^®^Phyt treatment (*p =* 0.007). In the jejunum, a significantly greater crypt depth but narrower villus width were recorded under both the Anta^®^Phyt and PreAcid treatments compared to CON (*p =* 0.026 and *p =* 0.007, respectively), and treatments had no effect on the ileum indices (*p >* 0.05). The gut mucosa is the first tissue to contact dietary bioactive compounds. Therefore, mucosal morphostructures are an indicator of intestinal integrity. In general, a nondisturbed mucosa is characterized by longer and wider villi, which ensure the maximal absorptive surface area and improved uptake of nutrients [29]. Thus, the application of the investigated additives in the broiler diets in the present study largely did not disturb the proper development of the gut. One potential concern is that villus area was reduced in some cases due to a decreased villus width; however, this change was not manifested in the BW of the birds, indicating that it was not sufficient to compromise bird growth. Nevertheless, this response contrasts with the findings of a study by Hashemi et al. [30], who reported increases in the height and surface area of villi in broilers fed an acidifier-supplemented diet. In line with this, Jamroz et al. [6] showed villus-related protective properties of plant extract supplementation in broilers. Interestingly, in the present study, feeding the broilers the diet containing the butyric acidifier resulted in deeper crypts in birds at 35 and 42 days of age, which are indicative of a higher rate of enterocyte cell renewal. According to Deepa et al. [31], this could be attributed to the antimicrobial properties of butyric acid. On the other hand, Emami et al. [32] demonstrated beneficial actions of dietary organic acids (formic and propionic acids) on the stimulation of intestinal mucosal growth in broilers, which was suggested to be due to trophic effects stimulating mucosal cell proliferation. One of the clinical signs of clostridial enteric disease is thin-walled, friable intestines [33]. In this regard, a beneficial action of feeding the broilers the experimental diets was evident in the jejunum of 35-day-old birds, which exhibited a thicker wall. These findings seem to confirm that both additives may exhibit some antibacterial activity against *Clostridial enteritis*.

## 4. Conclusions

It might be concluded that the feeding of birds with diets containing Anta^®^Phyt or PreAcid additives beneficially affected the activity of the cecal microflora indicated by SCFA concentrations. The positive effect was associated with higher concentrations of acetic acid and butyric acid in the cecal digesta in 28-d-old birds and a beneficial shift in these acids in the SCFA profile in 35-d-old birds. The feeding of the experimental diets maintained normal bacterial fermentation process in the cecal digesta of the birds at these ages. It seems that the effects of both additives on the SCFA concentrations in the cecal digesta were more pronounced in younger birds. Dietary supplementation with Anta^®^Phyt and PreAcid additives had no adverse effect on the activity of the cecal bacteria. Our findings indicate that the two additives did not disturb gut development as chicken growth proceeded and had some beneficial effects promoting intestinal wall strengthening and cell turnover.

## Figures and Tables

**Table 1:** Effect of dietary treatments on short-chain fatty acid (SCFA) concentrations in the cecal digesta of chickens at 28, 35, and 42 days of age.

Indices	Dietary Treatments ^1^			SEM	*p*-Value
	CON	Anta^®^Phyt	PreAcid		
	Birds at 28 days of age				
SCFA concentrations (μmol/g)					
Acetic	60.8 ^ab^	56.4 ^a^	73.2 ^b^	2.90	0.042
Propionic	4.47	3.42	5.04	0.353	0.165
Iso-butyric	0.69	0.70	0.92	0.068	0.320
Butyric	15.4 ^ab^	12.80 ^a^	19.7 ^b^	1.160	0.040
Iso-valeric	0.49	0.53	0.80	0.071	0.153
Valeric	1.03 ^a^	0.97 ^a^	1.36 ^b^	0.071	0.049
Sum of SCFAs	82.9 ^ab^	74.9 ^a^	101.0 ^b^	4.113	0.022
SCFA profile (% of total SCFAs)					
Acetic	74.0	75.4	72.5	0.765	0.316
Propionic	5.46	4.70	5.04	0.363	0.719
Butyric	17.9	16.8	19.4	0.775	0.391
	Birds at 35 days of age				
SCFA concentrations (μmol/g)					
Acetic	71.4	57.9	61.6	3.524	0.277
Propionic	4.36	3.56	4.09	0.318	0.585
Iso-butyric	0.91	0.79	1.01	0.067	0.432
Butyric	16.8	20.4	15.9	1.057	0.186
Iso-valeric	0.69	0.76	0.91	0.064	0.401
Valeric	1.45	1.58	1.66	0.077	0.567
Sum of SCFAs	95.7	84.9	85.2	4.467	0.555
SCFA profile (% of total SCFAs)					
Acetic	74.5 ^c^	68.4 ^a^	71.4 ^b^	0.675	0.001
Propionic	4.46	4.36	4.98	0.258	0.585
Butyric	17.7 ^a^	23.4 ^b^	18.8 ^a^	4.767	0.006
	Birds at 42 days of age				
SCFA concentrations (μmol/g)					
Acetic	54.9	55.1	47.2	2.351	0.300
Propionic	4.80	4.30	3.72	0.285	0.297
Iso-butyric	1.15	0.82	0.84	0.064	0.061
Butyric	12.9	12.6	11.9	0.849	0.888
Iso-valeric	1.05	0.70	0.72	0.084	0.166
Valeric	1.46	1.28	1.27	0.080	0.562
Sum of SCFAs	76.3	74.8	65.7	3.252	0.357
SCFA profile (% of total SCFAs)					
Acetic	72.0	73.8	72.2	0.593	0.426
Propionic	6.46	5.82	5.88	0.328	0.686
Butyric	16.6	16.5	17.2	0.603	0.878

^1^ Birds were fed a control diet (CON); the control diet supplemented (on top) with phytogenic ingredients from hops, licorice and arabic gum (Anta^®^Phyt); or a diet supplemented with a mixture of a free butyrate acidifier and prebiotic gluconic acid (PreAcid). Data are the means ± SEMs of 12 replicates per treatment. ^abc^ Means within a row without a common superscript differ significantly (*p <* 0.05).

**Table 2:** Effect of dietary treatments on morphological indices of the duodenal, jejunal, and ileal mucosa in 28-d-old chickens.

Indices	Dietary Treatments ^1^			SEM	*p*-Value
	CON	Anta^®^Phyt	PreAcid		
Duodenum					
Villus height, µm	1728.2	1646.1	1615.8	54.04	0.692
Crypt depth, µm	197.9	186.3	179.2	5.82	0.431
Villus width, µm	172.4	166.8	179.8	4.79	0.549
Wall thickness, µm	153.0	165.6	135.7	6.29	0.15
Villus area, µm^2^	971.4	893.1	937.7	42.32	0.761
Villus height to crypt depth	9.10	9.03	9.11	0.36	0.996
Jejunum					
Villus height, µm	1047.8	1061.8	955.5	36.48	0.471
Crypt depth, µm	171.3	161.3	152.1	4.69	0.257
Villus width, µm	155.7	137.3	144.3	5.41	0.364
Wall thickness, µm	126.6	134.1	116.7	4.62	0.314
Villus area, µm^2^	532.8	471	440.6	26.48	0.37
Villus height to crypt depth	6.25	6.58	6.40	0.227	0.84
Ileum					
Villus height, µm	703.7	728.9	656.9	18.7	0.279
Crypt depth, µm	168.3	170	152.4	3.8	0.101
Villus width, µm	178.4	166.1	174.0	5.0	0.607
Wall thickness, µm	138.6	143.9	130.6	4.3	0.453
Villus area, µm^2^	425.1	401.1	381.5	16.2	0.564
Villus height to crypt depth	4.24	4.28	4.33	0.1	0.934

^1^ Birds were fed a control diet (CON); the control diet supplemented (on top) with phytogenic ingredients from hops, licorice and arabic gum (Anta^®^Phyt); or a diet supplemented with a mixture of a free butyrate acidifier and prebiotic gluconic acid (PreAcid). Data are the means ± SEMs of 12 replicates per treatment.

**Table 3:** Effect of dietary treatments on morphological indices of the duodenal, jejunal, and ileal mucosa in 35-d-old chickens.

Indices	Dietary Treatments ^1^			SEM	*p*-Value
	CON	Anta^®^Phyt	PreAcid		
Duodenum					
Villus height, µm	1889.4	1860.9	1841.4	32.76	0.836
Crypt depth, µm	198.8 ^a^	196.3 ^a^	217.4 ^b^	3.01	0.005
Villus width, µm	162.3 ^c^	136.5 ^a^	148.8 ^b^	2.63	0.001
Wall thickness, µm	179.9	182.1	190.6	3.49	0.434
Villus area, µm^2^	982.2 ^b^	820.9 ^a^	877.8 ^a^	23.14	0.011
Villus height to crypt depth	9.50	9.52	8.49	0.21	0.051
Jejunum					
Villus height, µm	1221.2	1264.2	1274.4	31.11	0.774
Crypt depth, µm	166.4 ^a^	162.3 ^a^	178.3 ^b^	2.06	0.001
Villus width, µm	154.2 ^b^	130.2 ^a^	126.6 ^a^	2.68	0.001
Wall thickness, µm	149.4 ^a^	158.0 ^b^	162.8 ^b^	1.76	0.004
Villus area, µm^2^	613.9	478.6	518.7	23.38	0.057
Villus height to crypt depth	7.32	7.07	7.13	0.271	0.931
Ileum					
Villus height, µm	882.8	851.6	890.9	23	0.778
Crypt depth, µm	171.9	164.8	172.8	2.12	0.246
Villus width, µm	149.3 ^b^	143.9 ^ab^	140.9 ^a^	1.44	0.048
Wall thickness, µm	169.9	164.2	164.6	3.84	0.804
Villus area, µm^2^	430.6	403.5	406.0	10.92	0.539
Villus height to crypt depth	5.19	5.13	5.10	0.13	0.982

^1^ Birds were fed a control diet (CON); the control diet supplemented (on top) with phytogenic ingredients from hops, licorice, and arabic gum (Anta^®^Phyt); or a diet supplemented with a mixture of a free butyrate acidifier and prebiotic gluconic acid (PreAcid). Data are the means ± SEMs of 12 replicates per treatment. ^ab^ Means within a row without a common superscript differ significantly (*p <* 0.05).

**Table 4:** Effect of dietary treatments on morphological indices of the duodenal, jejunal, and ileal mucosa in 42-d-old chickens.

Indices	Dietary Treatments ^1^			SEM	*p*-Value
	CON	Anta^®^Phyt	PreAcid		
Duodenum					
Villus height, µm	1822.8	1738.1	1879.7	72.47	0.731
Crypt depth, µm	219.8	209.9	210.6	4.44	0.617
Villus width, µm	173.0 ^b^	146.6 ^a^	161.5 ^ab^	3.57	0.007
Wall thickness, µm	192.5	209.7	189.4	7.98	0.542
Villus area, µm^2^	1023.6	822.3	982.8	50.76	0.236
Villus height to crypt depth	8.41	8.35	9.15	0.323	0.545
Jejunum					
Villus height, µm	1296.5	1342.6	1340.5	33.11	0.823
Crypt depth, µm	173.0 ^a^	189.9 ^b^	195.0 ^b^	3.62	0.026
Villus width, µm	147.4 ^b^	122.6 ^a^	126.9 ^a^	3.56	0.007
Wall thickness, µm	180.3	161.7	161	6.88	0.442
Villus area, µm^2^	636.3	528.5	546.8	19.44	0.053
Villus height to crypt depth	7.69	7.09	6.88	0.242	0.365
Ileum					
Villus height, µm	786.2	908.1	840.1	37.68	0.423
Crypt depth, µm	161.6	177.4	170.9	3.89	0.242
Villus width, µm	135.2	135.2	134.8	4.04	0.999
Wall thickness, µm	182.8	179.1	172.4	8.39	0.887
Villus area, µm^2^	342.9	397.6	365.3	15.65	0.368
Villus height to crypt depth	4.93	5.14	4.70	0.203	0.690

^1^ Birds were fed a control diet (CON); the control diet supplemented (on top) with phytogenic ingredients from hops, licorice and arabic gum (Anta^®^Phyt); or a diet supplemented with a mixture of a free butyrate acidifier and prebiotic gluconic acid (PreAcid). Data are the means ± SEMs of 12 replicates per treatment. ^ab^ Means within a row without a common superscript differ significantly (*p <* 0.05).
